# Surface hemocompatible modification of polysulfone membrane *via* covalently grafting acrylic acid and sulfonated hydroxypropyl chitosan

**DOI:** 10.1039/c8ra10573a

**Published:** 2019-02-21

**Authors:** Ming-Ming Tu, Jing-Jie Xu, Yun-Ren Qiu

**Affiliations:** College of Chemistry and Chemical Engineering, Central South University Changsha 410083 PR China csu_tian@csu.edu.cn +86-13507479124

## Abstract

In this study, acrylic acid (AA) and sulfonated hydroxypropyl chitosan (SHPCS) were covalently grafted on the PSf membrane surface to improve its hemocompatibility. First, the modified AA-PSf membrane was obtained through the Friedel–Craft reaction between acrylic acid and the PSf membrane surface. Then, the modified SHPCS-AA-PSf membrane was prepared by grafting SHPCS onto the AA-PSf membrane surface *via* the dehydration acylation of the carboxyl group of the AA-PSf membrane with the amino group of SHPCS. ATR-FTIR and XPS measurements confirmed that the –COOH group and SHPCS were successfully grafted onto the surface of the PSf membrane. The modified PSf membranes showed suppressed platelet adhesion and lower protein adsorption (161 μg cm^−2^) compared with the pristine PSf membrane (341 μg cm^−2^). Hemocompatibility testing showed that modified membrane materials had a prolonged clotting time, plasma recalcification time (PRT), activated partial thromboplastin time (APTT), thrombin time (TT), and prothrombin time (PT). All of these results indicated that the surface modification of the PSf membrane with acrylic acid and SHPCS had good hemocompatibility and anticoagulant property.

## Abstract 2

In this study, acrylic acid (AA) and sulfonated hydroxypropyl chitosan (SHPCS) were covalently grafted on the PSf membrane surface to improve its hemocompatibility.

## Graphical abstract

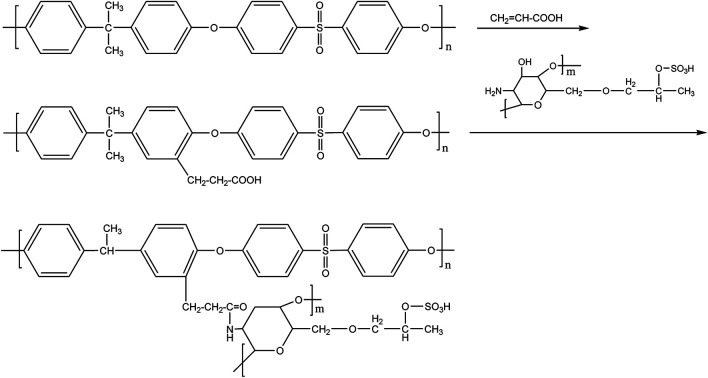

## Introduction

Polysulfone (PSf) membrane materials have been widely acknowledged and commercially used in many fields such as blood purification and hemodialysis over the last few years.^1^ 71% of hemodialysis membranes use PSf as materials, which had been investigated by the market data analysis of the Fresenius Medical Company in 2010.^2^ Compared with the first generation of membrane materials such as cellulose and its ramifications, PSf, as a second-generation membrane material, has higher flux and can be more effective in the removal of “middle” molecules.^3–5^ Besides, PSf is widely used because of its great mechanical strength, thermal stability, chemical resistance, as well as its membrane-forming properties.^6,7^ Nevertheless, PSf material is hydrophobic by nature; when it is used as blood-contacting membranes, a series of bio-responses such as apparent protein adsorption, platelet adhesion, and the activation of the clotting enzyme will occur, which directly leads to thrombogenesis.^8,9^ Therefore, it is reasonable and necessary to modify pristine PSf to ameliorate its hemocompatibility.

Heparin, acknowledged as an effective anticoagulant agent, has been widely used to prevent the formation of coagulation and to improve the hemocompatibility of blood-contacting membranes. However, heparin treatment is expensive, which may limit its large-scale use to some extent.^10,11^ Herein, numerous attempts have been focused on developing heparin-mimicking materials or incorporating heparin-mimetic structures into membranes for the replacement of heparin to improve the hemocompatibility and tissue compatibility of the materials.^12,13^ Qin *et al.*^14^ provided a highly efficient, convenient and universal protocol for the blood-compatible modification of polyethersulfone (PES) membranes *via* the *in situ* cross-linked copolymerization of 2-hydroxyethyl methacrylate (HEMA) and acrylic acid (AA) in PES solutions, and the results indicated that the modified membranes showed improved hydrophilicity, good blood anticoagulant and antifouling properties after grafting HEMA and AA. Kim *et al.*^15^ prepared sulfonated polyethersulfone by the heterogeneous method with chlorosulfonic acid; the modified membrane with a –SO_3_H group could reduce fouling and exhibited good blood compatibility. Lijing Zhu *et al.*^16^ fabricated PSf hemodiafiltration membranes by the *in situ* cross-linked polymerization of vinyl pyrrolidone (VP) and vinyltriethoxysilane (VTEOS) in PSf solutions and the non-solvent induced phase separation (NIPS) technique. Ran *et al.*^17^ used vinyl pyrrolidone (VP) and methyl methacrylate (MMA) to modify the PES membrane *via* the blending method, and the blood compatibility, ultrafiltration and antifouling properties of the biomaterials were improved.

A lot of work have been done in terms of fabricating the modified membrane by grafting heparin-mimicking or heparin-like polymeric materials, which contain functional polar groups such as the hydroxyl group (–OH), carboxyl group (–COOH) and the sulfonic acid group (–SO_3_H),^18–20^ onto membrane surfaces. This could be considered as an effective way to improve hemocompatibility, as these materials have similar specific functional groups to heparin.^21^ Among these modified methods such as coating, blending, grafting, *in situ* cross-linked copolymerization and layer-by-layer assembly methods,^22–24^ grafting is commonly considered as a useful and stable method compared with other methods. Chitosan (CS) has both amino and hydroxyl groups and has been identified as a non-toxic, biodegradable, cell compatible and biocompatible material.^25–27^ Yang *et al.*^28^ grafted CS oligomer onto the polysulfone membrane *via* ozone-treatment, and COS-coupled PSf membranes showed a stronger biocidal effect for bacteria than untreated PSf membranes. Filiz *et al.*^29^ synthesized and modified PU *via* covalent immobilization of CS to improve its antibacterial properties. Liu *et al.*^30^ covalently immobilized citric acid (CA) and CS onto polyurethane (PU) materials followed by blending with PES; the modified membrane had good anticoagulant and antibacterial properties. Nowadays, CS has been utilized and exploited widely and deeply. M. Huo *et al.*^31^ investigated the synthesis and anticoagulant activity of three different kinds of CS derivatives of similar structure including hydroxybutyl chitosan, hydroxypropyl chitosan sulfates, and quaternary ammonium chitosan sulfates. Fang *et al.*^32^ prepared sulfonated hydroxypropyl chitosan (SHPCS), which is a heparin-like material with excellent hemocompatibility. In our previous study, SHPCS was grafted onto the PSf membrane material by the Schiff-base reaction. The –CH_2_Cl groups were grafted onto PSf and then transformed into the PSf-Cl membrane through a phase-inversion technique, followed by immersion in ethylenediamine to introduce the amino, so that the sulfonated hydroxypropyl chitosan could be eventually grafted from the modified membrane with glutaraldehyde as the “bridge”.^33^ However, the operation conditions are not moderate, and the synthesis process is time-consuming and complicated. Therefore, developing an efficient way to modify the PSf membrane with heparin-mimicking SHPCS on a large-scale remains highly desirable. We proposed that acrylic acid (AA) was firstly grafted onto the PSf membrane surface, which could allow –COOH to further react with heparin-like SHPCS and improve the hemocompatibility of the membrane. Compared with the traditional Schiff-base reaction, the modification of the PSf membrane *via* covalently grafting acrylic acid and SHPCS is simpler and more straightforward.

In order to graft –COOH onto the PSf membrane, PSf was used as the base. Acrylic acid was grafted onto the membrane surface by a F–C reaction between PSf membrane and acrylic acid, and the modified membrane was termed the AA-PSf membrane. Afterwards, the modified membrane (SHPCS-AA-PSf) can be obtained by dehydration acylation of the carboxyl group of the AA-PSf membrane with the amino group of the SHPCS. Attenuated total reflection-Fourier transform infrared (ATR-FTIR) spectroscopy, XPS, scanning electron microscopy (SEM), and water contact angle (WCA) measurements were applied to confirm the chemical components, surface morphology, and hydrophilicity of the modified PSf membranes. The hemocompatibility of the modified membrane was investigated by measuring the contact angle (WCA) and blood compatibility (protein adsorption, platelet adhesion, plasma recalcification time (PRT), hemolysis, APTT, PT and TT).

## Methods

### Materials

Polysulfone (average *M*_n_: 22 000) and tin(iv) chloride were purchased from Sigma, USA. Chitosan (viscosity: 100–200 mPa s) with a degree of deacetylation of about 95% was obtained from Aladdin Industrial Corporation. Sodium dodecyl sulfate (SDS), sodium hydroxide (NaOH; AR), propylene oxide (AR), formamide (AR), bovine serum albumin (BSA; AR) and sodium chloride (AR) were purchased from the Sinopharm Chemical Reagent Company, China. *N*,*N*-Dimethylacetamide (DMAC) used as the solvent was obtained from the Guangdong Guanghua Sci-Tech Company, China. Isopropanol (AR) and acetone were purchased from the Chengdu Kelong Chemical Reagent Company, China. Chlorosulfonic acid (CP) was acquired from Beijing Mashi Fine Chemicals Company, China. The MD44 dialysis bag (diameter: 28 mm; molecular weight cut-off: 8000 dalton) was purchased from the Shanghai Leibusi Company.

### Modification of the PSf membrane

#### PSf membrane was modified using acrylic acid

**Fig. 1:**
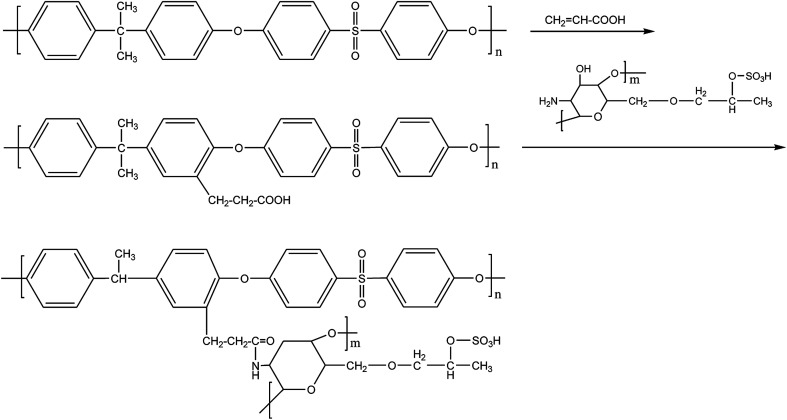
The steps of the modification of the PSf membrane.

PSf flat-sheet membranes were prepared according to the method described by Xin Tian *et al.*^34^ The 18 wt% PSf was solved in the DMAC solvent with oscillation until a clear homogeneous solution was obtained. Afterward, the mixture was placed down to get rid of any bubbles at ambient temperature, cast onto a glass plate waiting for 10 s at room temperature, and then immersed in distilled water. The PSf flat-sheet membranes were obtained through the immersion precipitation phase inversion method and were cut into 1.0 × 1.0 cm^2^ pieces for later use after being dried under vacuum (Fig. 1).

Firstly, the PSf flat-sheet membranes were washed with NaOH solution (0.1 mol L^−1^) and ultra-pure water. After being dried under vacuum, the PSf was subsequently incubated into a round-bottom flask containing acrylic acid (14.5 mol L^−1^). Simultaneously, 2.86 g of tin tetrachloride and 5 mL of phosphoric acid (19.2 mol L^−1^) were added as an activator under slight stirring at 35 °C for a certain amount of time (30 min, 45 min, 60 min, 75 min, 90 min). Thus, the carboxyl groups were introduced to the PSf surface *via* the Friedel–Crafts alkylation reaction. In the end, the modified membranes grafted with acrylic acid, which were termed AA-PSf, were washed clearly with ultra-pure water to remove any unreacted substances. Furthermore, the surface grafting density of carboxyl groups was analyzed by the amount of toluidine blue O (TB) dye adsorption.^35^ The carboxyl group on the membrane could form a complex with toluidine blue O (TB) dye at pH 10; the complex dye molecules were then desorbed with 50 wt% acetic acid solution. Afterwards, the absorbance of the supernatant was determined at 633 nm by UV spectrophotometry.

#### AA-PSf membrane was modified by SHPCS

These AA-PSf membranes were initially incubated in EDC solution (0.01 mol L^−1^) and citric acid buffer at 4 °C for 24 h. Then, the membranes were immersed in 25 mL of SHPCS solution with a few drops of acetic acid at 25 °C for a certain number of hours (five groups: 1 h, 3 h, 7 h, 11 h, 15 h, 18 h, 20 h). It worth noting that SHPCS was synthesized as described in our previous study.^33^ After being washed several times with ultra-pure water and dried in a vacuum, the modified membranes grating with SHPCS, which were termed SHPCS-AA-PSf, were finally obtained. The grafting yield (mg cm^−2^) was calculated as follows:
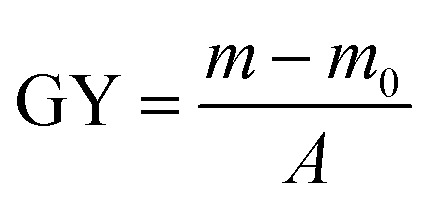
where GY (mg cm^−2^) is the grafting yield, *m*_0_ (mg) and *m* (mg) represent the weight of the dried membrane before and after grafting polymerization, respectively, and *A* (cm^2^) is the area of the membrane. All the results are the average values.

### Characterization of the SHPCS-AA-PSf membrane

The attenuated total reflectance-Fourier-transform infrared (ATR-FTIR) spectra for modifying the membrane surfaces were measured using a Fourier-transform infrared spectrometer (Nicolet6700, USA). Elemental analyses of the modified membranes were performed using an ESCALAB 250Xi XPS instrument (Thermo Scientific, USA). The hydrophilicity of the membrane surface was evaluated using a contact angle goniometer (JC-2000D1, China) equipped with video capture on the basis of contact angle measurement. For the static contact angle measurements, a total of 3 μl of distilled water was dropped on the air-side surface of the membrane at room temperature, and the contact angle was measured after 20 s. At least five measurements were averaged to obtain a reliable value. A scanning electron microscope (SEM, JSM-IT300LA Japan) was used for the morphology observation of the membrane cross-sections. All samples were dried overnight in a vacuum oven at room temperature and then quenched with liquid nitrogenous gas, attached to the sample supports and coated with a gold layer.

### Blood compatibility

#### Protein adsorption

For the protein adsorption experiments, the method is similar to those described by Zhu *et al.*^36^ The membranes with an area of 1 × 1 cm^2^ were incubated in phosphate buffer solution (PBS) for 24 h before use. Afterwards, the sample was immersed in the protein solution, which contains BSA and PBS (pH = 7.4) with a concentration of 1 mg/1 mL, for 2 h at 37 °C. Subsequently, the sample was rinsed slightly with PBS solution several times and then immersed in washing aqueous solutions (2 wt% sodium dodecyl sulfate (SDS), 0.05 M NaOH, 37 °C with agitation for 2 h) to remove the adsorbed protein. More than 95% of the adsorbed protein could be eluted into the SDS solution, and the amount of protein could be evaluated using the ultraviolet absorption method.

#### Platelet adhesion

This method is similar to those described by Zhang *et al.*^37^ Healthy fresh blood samples (man, 25 years old) were collected by vacuum tubes (2 mL G9NC2002, sodium citrate to blood ratio 1 : 9), which were centrifuged at 1000 rpm for 15 min to obtain platelet-rich plasma (PRP). Then, the membrane (1 cm × 1 cm) was immersed in PBS solution at 37 °C for 1 h, followed by the removal of PBS solution, and the addition of 1 mL of fresh PRP. Next, the membrane was incubated in PRP at 37 °C for 2 h and subsequently rinsed slightly with PBS solution three times. Afterward, the membrane was treated with 2.5 wt% glutaraldehyde in PBS solution at 4 °C for 1 day. After being washed with PBS solution, the sample was immersed through a series of graded alcohol-PBS solutions (0%, 25%, 50%, 75% and 100%) and graded isoamyl acetate–alcohol solutions (25%, 50%, 75% and 100%) for 15 min, respectively. Afterwards, the membrane was freeze-dried, and the adherent platelet on the membrane was observed by SEM.

#### Plasma recalcification time (PRT)

The membrane (1 cm × 1 cm) was immersed in the PBS solution and equilibrated at 37 °C for 1 h. Then, 0.5 mL of fresh PPP obtained by centrifugation at 3000 rpm for 15 min was introduced and afterward incubated statically at 37 °C. Subsequently, 0.5 mL of 25 mM CaCl_2_ aqueous solution was added, and the plasma solution was monitored for clotting by manually dipping a stainless-steel hook coated with silicone into the solution to detect fibrin threads. Clotting times were recorded at the first sign of fibrin formation on the hook. The test was repeated three times for each sample to obtain a reliable value.

#### Hemolysis assay

The membrane sample (1 cm × 1 cm) was immersed in normal saline at 37 °C for 1 h at first. The entirety of the blood was diluted with the addition of normal saline, and the dilute solution was introduced into the tube containing the membrane sample. Besides, positive and negative controls were prepared by adding 2 mL of blood to 12 mL of ultra-pure water and normal saline, respectively. After that, all the samples were equilibrated in dilute solutions at 37 °C for 3 h and then centrifuged at 500 g for 10 min. The percent hemolysis was calculated by measuring the absorbance of the supernatant solution at 545 nm using a UV-vis spectrophotometer. The hemolysis ratio (HR) was calculated as follows:
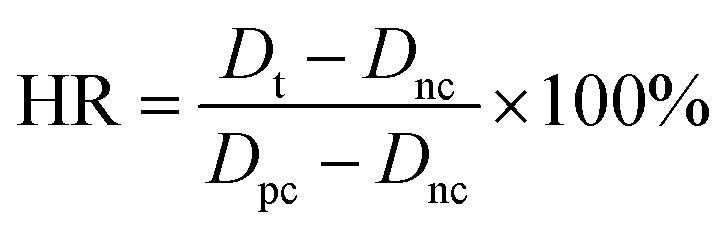
where *D*_t_ is the absorbance of the test sample, *D*_nc_ is the absorbance of the negative control and *D*_pc_ is the absorbance of the positive control.^33^

#### APTT, PT and TT

The membrane (1 cm × 1 cm) was washed with PBS three times and dried. Then, the sample was immersed in 1 mL of PPP at 37 °C for 1 h. The APTT, PT and TT were obtained by a fully automatic blood coagulation detector (CA-7000) in the Fourth Hospital of Changsha.

### Antibacterial tests

#### Medium preparation and sterilization

Nutrient broth liquid medium and nutrient agar solid medium were prepared separately. Afterwards, the medium, PBS, and all equipment used for the antibacterial experiments were placed in an autoclave, sterilized at 121 °C for 20 min, and stored in a clean bench. At the same time, the nutrient agar solid medium was cooled to about 50 °C, and an appropriate amount of liquid was evenly poured into the culture dish to prepare a nutrient agar plate for use.

#### Antibacterial experiment

The *Pseudomonas aeruginosa* strain (*P. aeruginosa*) was inoculated into a test tube containing 10 mL of nutrient broth liquid medium and cultured in a biochemical incubator at a temperature of 37 °C for 18–24 h. After the bacteria were proliferated, the bacteria-containing liquid was dispensed into three centrifuge tubes and centrifuged at 3000 rpm for 10 min in a centrifuge to precipitate the bacteria in the lower layer while removing nutrients from the bacterial liquid. The bacterial pellet was then washed twice with PBS, and the bacteria were suspended in 10 mL of PBS. 100 μL of the bacterial suspension was placed on the surface of the PSf, AA-PSf and SHPCS-AA-PSf membranes; 100 μL of the bacterial suspension was placed in a centrifuge tube as the blank control group. Thereafter, all samples were placed in a biochemical incubator and incubated at 37 °C for 24 h. Next, all patch samples were transferred to a centrifuge tube, and 1 mL of PBS was added to all centrifuge tubes. The bacterial suspension was serially diluted, 100 μL of the dilution in each gradient was placed on a nutrient agar plate, and the bacterial solution was uniformly coated with a coating bar. Finally, all the nutrient agar plates were placed in a biochemical incubator at 18 °C for 18–24 h, and the number of viable bacteria in the original bacteria solution was estimated by counting the number of colonies (CFU) formed.

### Live subject statement

This study was performed with the approval of the Ethics Committee of Xiangya Medical College, Central South University, Changsha, China. All the experiments of blood compatibility were performed in accordance with the guidelines of the National Health Commission of China, and informed consent was obtained from all human participants in this study.

## Results and discussion

### PSf membrane was modified by acrylic acid and SHPCS

**Fig. 2:**
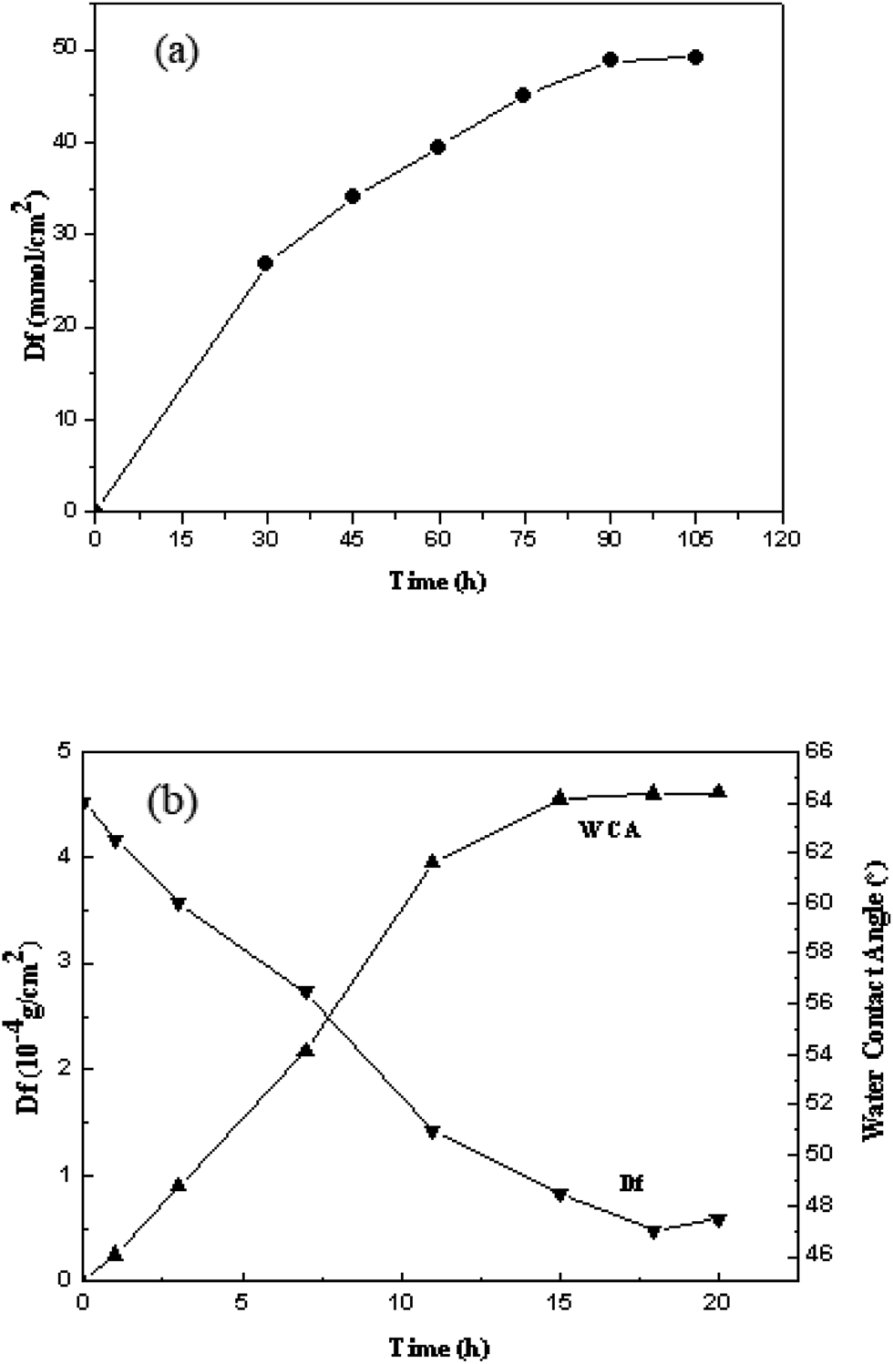
The grafting density of acrylic acid at different reaction times (a); the grafting density of SHPCS and the SHPCS-AA-PSf water contact angle at different reaction times (b).

As showed in Fig. 2(a), the grafting density of acrylic acid increased with the reaction time, but the reaction speed decreased with the reaction time. When the time reached 90 min, the greatest grafting density of 49.08 mmol cm^−2^ for acrylic acid was acquired. Nevertheless, the grafting density did not increase obviously from 90 min to 105 min, and the results showed that 90 min was the most proper reaction time.

The effects of different reaction times on the grafting density of SHPCS and the hydrophilicity of the modified SHPCS-AA-PSf were investigated. According to Elimelech *et al.*,^38^ the hydrophilic membrane can form a hydration layer on the surface and improve the antifouling performances. The water contact angle is a simple method to evaluate the hydrophilicity, which decreases with increasing hydrophilicity. As the reaction time continues to increase, the grafting density increased, and the contact angle of the membrane surface continuously decreases. The maximum grafting density of SHPCS reached 4.6 × 10^−4^ g cm^−2^, and the grafting reaction nearly reached saturation. The water contact angle remained around 47°.

### Surface characterization

#### ATR-FTIR analysis

**Fig. 3:**
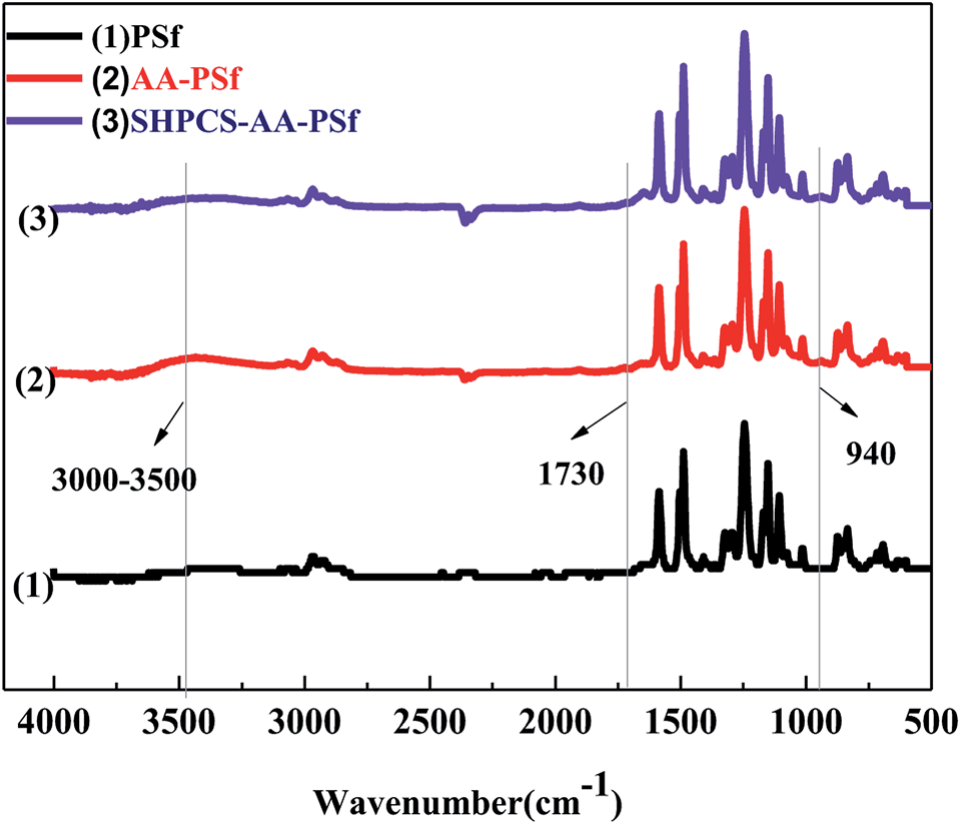
ATR-FTIR spectra of PSf, AA-PSf and SHPCS-AA-PSf membranes.

ATR-FTIR spectroscopy was used to track any changes of the functional groups on the surface of membranes in the process of modification, and the results were shown in Fig. 3. Compared with the infrared spectrum of the original PSf membrane, the AA-PSf membrane was stronger near 1730 cm^−1^. A new absorption peak of the modified SHPCS-AA-PSf appeared at 3500–3300 cm^−1^, which could be attributed to the characteristic peaks of the –OH and N–H stretching vibrations, indicating that the acrylic material was successfully grafted onto the membrane material. Among them, the –OH group is likely to come from SHPCS used to modify the membrane. At the same time, the SHPCS-AA-PSf membrane has a strong peak at 940 cm^−1^, which belongs to the C–N stretching signal, indicating that the –NH_2_ in SHPCS reacts with the oxidized PSf to form the amide bond. In addition, since the grafted material contains S=O, the signal of the modified membrane SHPCS-AA-PSf at the 1241 cm^−1^ signal of the S=O stretching characteristic peak is stronger than the original PSf and AA-PSf membrane. It was concluded that SHPCS was covalently grafted onto the surface of the membrane.

#### XPS analysis

**Fig. 4:**
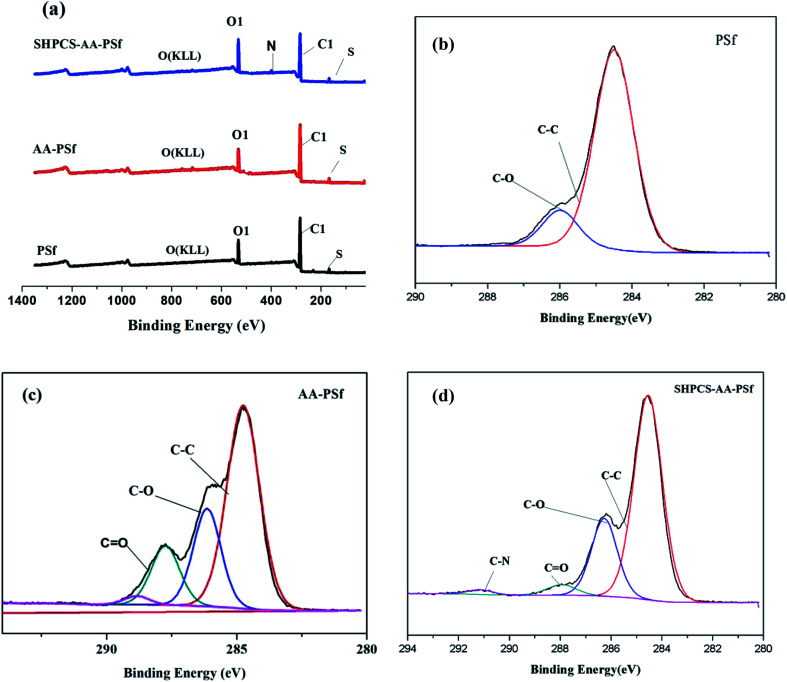
XPS spectra of PSf, AA-PSf, and SHPCS-AA-PSf membranes (a); spectra analysis of the PSf membrane (b), AA-PSf membrane (c) and SHPCS-AA-PSf membrane (d).

To further confirm whether acrylic acid was grafted onto the PSf membrane surface, and if SHPCS was introduced into the polysulfone membrane surface, XPS was used in this experiment to test the composition of chemical elements on the surface of the PSf membrane, AA-PSf membrane and SHPCS-AA-PSf membrane. The characterization results are shown in Fig. 4. The N1s signal peak of 399.58 eV only appears at the SHPCS-AA-PSf, showing that SHPCS was grafted onto the surface of the PSf membrane.

From the C1s peak plots in Fig. 4, it can be seen that C=O appears on the modified AA-PSf spectrum and C–N and C=O appear on the modified SHPCS-AA-PSf spectrum, which are consistent with the elemental analysis results presented in Table 1, Fig. 2 and 3. In the analysis of elemental content in Table 1, the N content in the original PSf membrane was 0, and C–N and C=O did not appear. After the acrylic acid was grafted, C=O appeared, and the content was 7.93%. After modification by SHPCS, C–N and C=O appeared on the surface of the SHPCS-AA-PSf membrane material, the contents of which were 0.98% and 2.43%, respectively, and the content of the N element also increased to 2.98%. The C–C content in the modified membrane SHPCS-AA-PSf decreased in comparison with C–C in PSf which was 48.67%, and the content of C=O increased to 17.04%. This is attributed to grafted SHPCS containing a large amount of O and N elements. Compared to the 2.57% of the S element before modification, the content of grafted SHPCS slightly increased to 3.02%. We know that the synthesized SHPCS contains sulfonic acid groups and the S element of the membrane material. The increase may originate from SHPCS. These results clearly demonstrate that SHPCS had been successfully covalently grafted onto the PSf membrane surface.

**Table 1:** Elemental analysis of the PSf membrane and PSf-SHPCS_b_ membrane surfaces

Samples	C (%)				N (%)	O (%)	S (%)
	C–C	C–O	C–N	C=O			
PSf	68.53	12.33	—	—	0	16.57	2.57
PSf-AA	45.70	16.93	—	7.93	0	27.37	2.07
PSf-SHPCS_b_	48.67	17.04	0.98	2.43	2.98	24.88	3.02

#### The cross-section morphologies of membranes

**Fig. 5:**
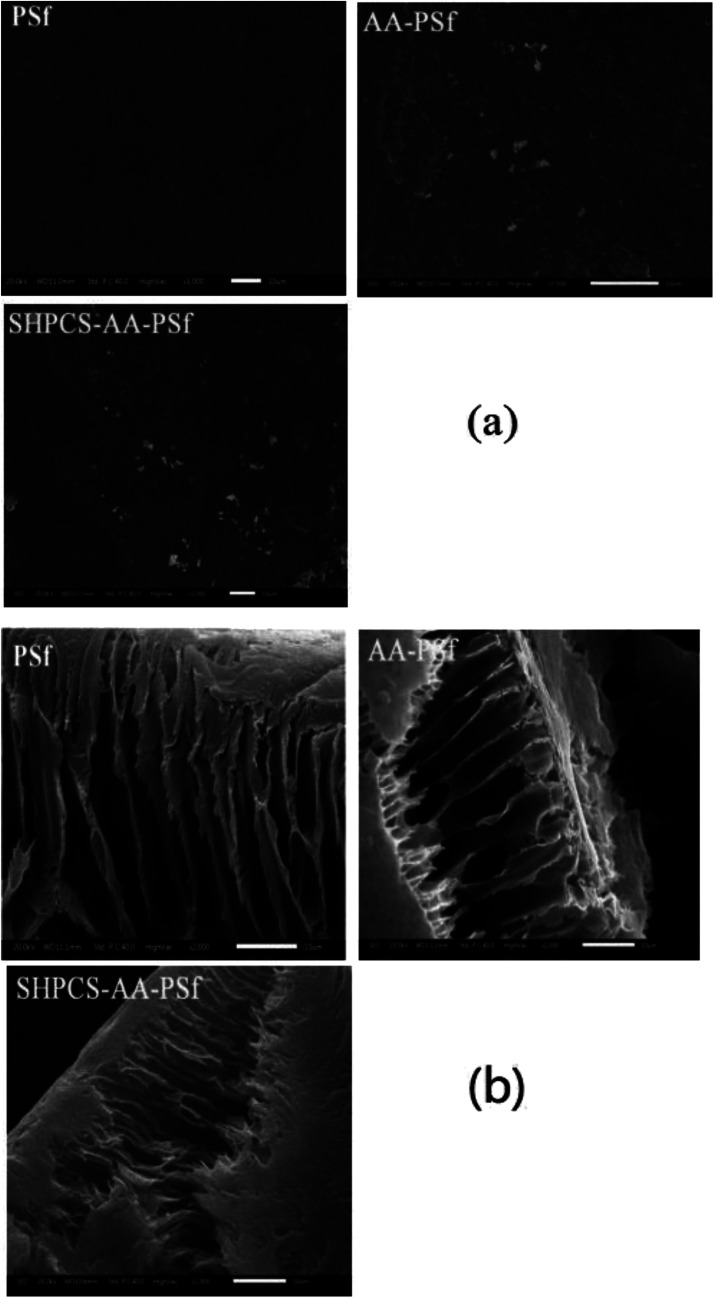
SEM images of PSf, AA-PSf and SHPCS-AA-PSf membrane surfaces (a); SEM photographs of PSf, AA-PSf and SHPCS-AA-PSf membrane cross-sections (b).

The SEM images of cross sections of the PSf, AA-PSf and SHPCS-AA-PSf membranes were shown in Fig. 5. As discussed previously, the popularity of the PSf membrane results from its various excellent properties, especially the high permeability for low-molecular-weight proteins, for the special morphology of the finger-like cross-sections consisting of a dense top-layer and porous sub-layer. It is worth noting that the surface SEM photograph clearly shows the change of the membrane surface from smooth material accumulation to the appearance of rough white micelles during the modification process, which is synchronous with changes in the properties of the membrane surface such as hydrophilicity. The asymmetric porous structure appeared in the cross-sectional SEM photographs of PSf, AA-PSf and SHPCS-AA-PSf membranes. Although the modification process increased the roughness of the membrane, the finger structure of the original unmodified PSf membrane was not subjected to major damage before and after modification.

#### Water contact angle analysis

**Fig. 6:**
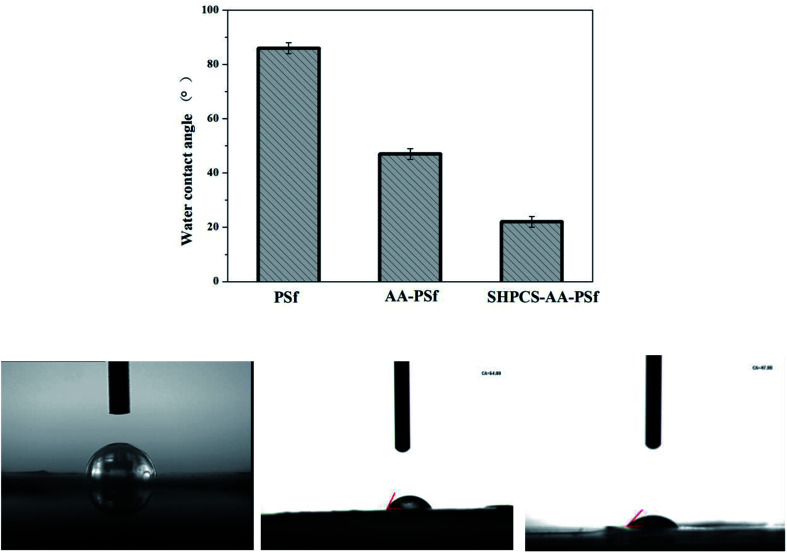
Surface contact angles of the PSf membrane, AA-PSf membrane and SHPCS-AA-PSf membrane.

Hydrophilic membrane materials have a high resistance to protein adsorption. The hydrophilic properties of the membrane during the modification process are determined by measuring the water contact angle on the surface of the membrane material.^39^ The results are shown in Fig. 6. The contact angle of the original PSf membrane was 86°. In comparison, the contact angle of the AA-PSf membrane grafted with acrylic acid decreased by 22°, indicating that the hydrophilicity of the membrane was somewhat improved. After grafting the highly hydrophilic heparinoid substance SHPCS, the contact angle of the modified membrane SHPCS-AA-PSf was significantly reduced to 47°, and the hydrophilicity change in this process can be clearly seen in Fig. 6. After modification, the hydrophilicity of the membrane improved, and the performance against protein contamination can be expected to improve.

### Blood compatibility of the modified membranes

#### Protein adsorption

**Fig. 7:**
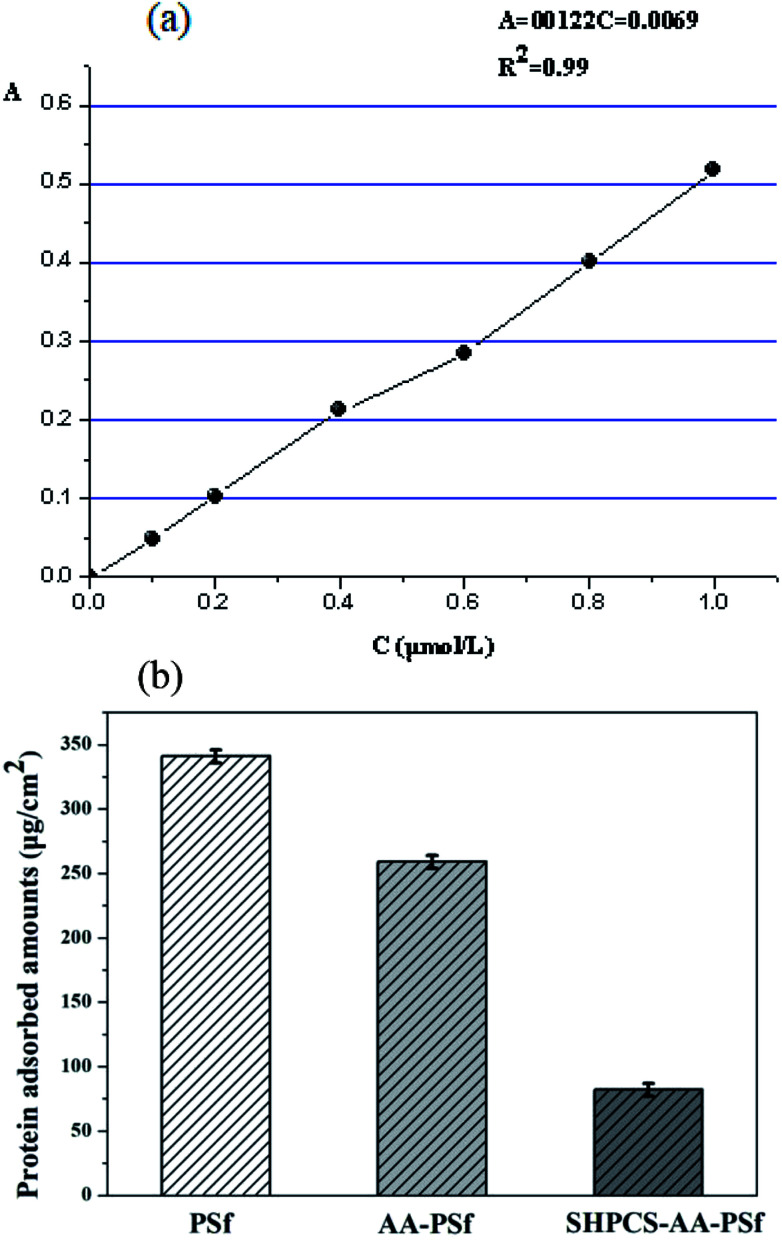
The standard curve of BSA absorbance (a); the adsorption of BSA on PSf, AA-PSf and SHPCS-AA-PSf membranes (b).

When the blood comes into contact with the surface of the material, it could induce platelet adhesion and activation of various coagulation pathways, leading to coagulation.^40^ Therefore, the amount of protein adsorption on the surface of the membrane is an important indicator of the blood compatibility of the membrane material. In this experiment, BSA was used to simulate the protein adsorption *in vitro*, for it has a similar molecular weight and Stokes radius to the human serum albumin. The results are shown in Fig. 7.

It can be seen that the modified AA-PSf and SHPCS-AA-PSf membranes were found to have lower BSA adsorption than the unmodified PSf membrane and that the amount of BSA adsorbed on the modified SHPCS-AA-PSf membrane is the lowest among the membranes. This may be because the SHPCS-AA-PSf membrane has both a sulfonic acid group and a carboxyl group in the heparin structure, greatly improving the hydrophilicity and resistance to protein adsorption. These results are also consistent with those of the hydrophilicity of the membranes discussed in previous sections. These also indicate that the BSA adsorption amount of the modified membrane was reduced and that the anti-protein contamination performance of the modified membrane, especially the SHPCS-AA-PSf modified membrane, was greatly improved.

#### Platelet adhesion

**Fig. 8:**
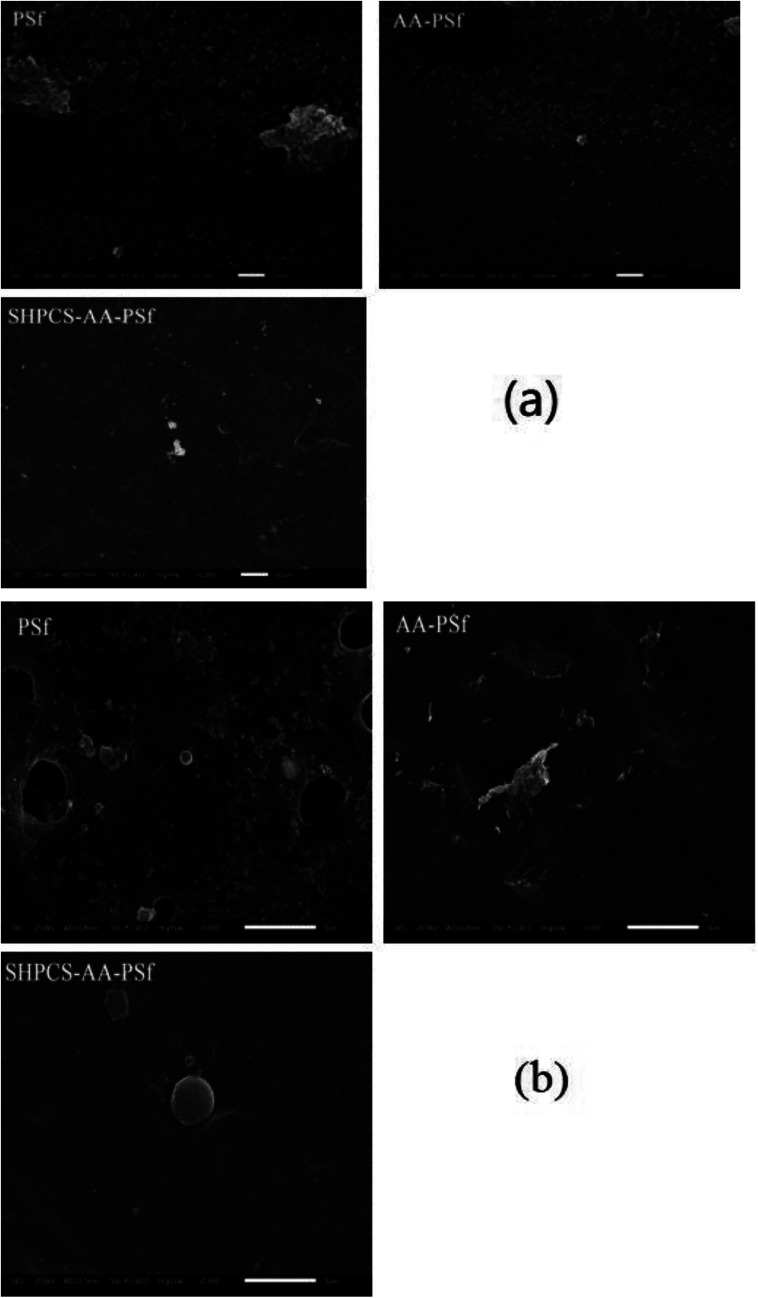
The SEM image of platelets adsorbed onto PSf, AA-PSf and SHPCS-AA-PSf membranes. Magnification: 1000× (a); magnification: 5000× (b).

The blood compatibility of the membrane material can be detected by performing an *in vitro* platelet adhesion test. After contacting the membrane material, on one hand, it adheres to the surface and activates to cause platelet aggregation. On the other hand, the activated platelets activate a variety of blood coagulation factors, causing coagulation on the surface of the membrane material. During this process, platelets accumulate and become flat or irregular in shape from the original circle, and a “pseudo-foot” extends around them. Therefore, we conducted scanning electron microscopy (SEM) of the number and shape of adherent platelets on the membranes (PSf, AA-PSf, and SHPCS-AA-PSf), and the results are shown in Fig. 8.

After 1000 scanning electron micrographs, it can be observed that a large amount of platelets accumulated on the original PSf membrane and appear irregularly arranged on the surface of the membrane. After grafting acrylic acid, the number of platelets can be seen to decrease, and after grafting SHPCS on the surface of the membrane, it was observed that the platelet distribution on the surface of the modified membrane was more dispersed and that the number of platelets decreased more than on the unmodified PSf membrane. The grafted SHPCS increased the blood compatibility of the material in terms of quantity and aggregation. In order to observe the morphology of the adhered platelets more clearly, 5000 SEM photographs were obtained. It can be observed from Fig. 7(b) that the platelets adhering to the surface of the PSf membrane have undergone irregular deformation and that the pseudopods have been extended. However, no deformation was observed on the surface of the SHPCS-AA-PSf membrane. This indicates that SHPCS plays a certain role in resisting platelet accumulation and deformation. These tests are consistent with BSA adsorption and other blood experiments, indicating a significant improvement in membrane blood compatibility after modification.

#### Plasma recalcification time (PRT)

**Fig. 9:**
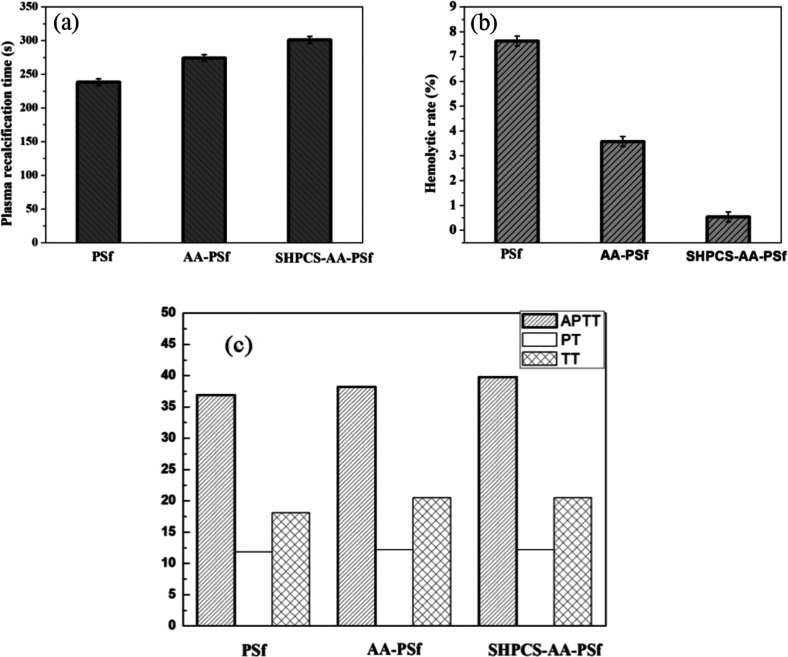
The plasma recalcification time of PSf, AA-PSf and SHPCS-AA-PSf membranes (a); the hemolysis rate of PSf, AA-PSf and SHPCS-AA-PSf membranes (b); the APTT, PT and TT of PSf, AA-PSf and SHPCS-AA-PSf membranes (c).

During endogenous coagulation, the activation of the coagulation factor XII causes a series of interactions between coagulation factors, resulting in the formation of thrombin by prothrombin. The plasma recalcification time (PRT) refers to the time required for calcium decalcification and calcium coagulation. It is often used to simulate endogenous coagulation processes *in vitro*, and the number of coagulation factors and anticoagulants affects plasma recalcification time (PRT). In order to further evaluate the anticoagulant properties of the modified membrane, we performed PRT tests on the original membrane and the modified membrane, and the results are shown in Fig. 9(a). It is not difficult to find that the plasma recalcification time of the PSf membrane is 238 s. When the surface of the membrane is grafted with SHPCS, the plasma recalcification time of the SHPCS-AA-PSf membrane reached 301 s. The change of PRT is obvious compared with the unmodified membrane. These results indicate that the modified membrane has a certain anticoagulant property.

#### Hemolysis assay

When red blood cells are damaged and release hemoglobin into the plasma, this phenomenon is called hemolysis. A large amount of hemolysis can directly induce coagulation, and the damage of the erythrocyte membrane may also cause platelet deformation and further lead to coagulation. Therefore, over the course of the experimental research, we used the hemolysis rate (HR) to evaluate the blood compatibility of the membrane materials.^41^ It can be seen from Fig. 9(b) that the hemolysis rate (HR) of the PSf membrane was 7.62%. After the grafting of acrylic acid by the Friedel–Craft reaction, the hemolysis rate of the AA-PSf membrane was 3.57%, which was lower than that of the unmodified PSf membrane. When SHPCS was grafted onto the surface of the membrane, the hemolysis rate (HR) of SHPCS-AA-PSf decreased sharply 0.53%, which was much lower than the international standard of ISO 10993; that is, the *in vitro* hemolysis (HR) experiment should be satisfied. Less than 5% is in line with the requirements of biomaterial blood testing, so it can be known that the modified SHPCS-AA-PSf membrane exhibits better blood compatibility than the unmodified PSf membrane.

#### APTT, PT and TT

APTT, TT and PT assays are widely used for the clinical screening of plasma abnormalities and the preliminary screening of anticoagulant drugs. Studies usually apply them to evaluate the *in vitro* antithrombotic ability of biological materials. APTT reflects the role of prothrombin, fibrinogen, factor V, and factor X in the endogenous coagulation pathway in blood and can be used to measure the efficacy of endogenous coagulation pathways. The TT blood test measures the thrombus formation time of plasma that has been added to thrombin and is often used as an *in vitro* plasma thrombin time measurement. PT is commonly used to evaluate exogenous coagulation pathways. The results of APTT, TT, and the PT of PSf, AA-PSf, and SHPCS-AA-PSf are shown in Fig. 9(c).

The difference in APTT, TT, and PT between the PSf (36.90 s, 18.10 s, and 11.80 s) and AA-PSf membrane (38.20 s, 18.90 s and 12.00 s) is slight. Compared with the unmodified membrane, the APTT of the modified equivalent was extended by 1.3 s, and the changes of TT and PT were 0.8 s. The APTT, TT, and PT measured by the heparin-like substance SHPCS in this experiment were 179 s, 62.10 s, and 29.20 s, respectively. These three data far exceeded the medical test reference values, indicating that SHPCS has a good anticoagulation effect. It can be seen from Fig. 8(c) that the APTT and TT of the modified SHPCS-AA-PSf membrane are larger than the corresponding values of the unmodified PSf membrane and that the PT does not change much. This is due to the grafting of the heparin-like substance SHPCS with anticoagulant properties, thereby affecting the endogenous coagulation pathway, which explains to some extent how the modified membrane also has an anticoagulant effect.

### Antibacterial test

**Fig. 10:**
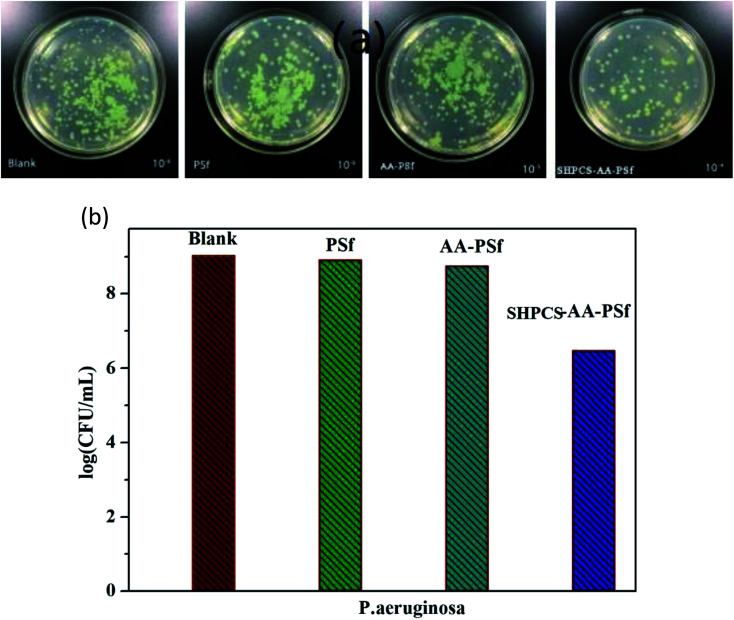
Images of *P. aeruginosa* under different culture conditions (a); effects of blank, PSf, AA-PSf and SHPCS-AA-PSf membrane on the survival number of *P. aeruginosa* (b).

In order to study the antibacterial properties of the modified PSf membrane, the antibacterial activity of all samples was tested using *Pseudomonas aeruginosa*, and the results are shown in Fig. 10. The logarithmic concentration of the bacterial between blank (9.03 CFU mL^−1^) and PSf membrane (8.92 CFU mL^−1^) showed nearly no significant difference. The AA-PSf membrane and SHPCS-AA-PSf membrane, the values reduced to 8.75 CFU mL^−1^ and 6.47 CFU mL^−1^, respectively. Compared to the pristine membrane, SHPCS-AA-PSf membrane showed good antibacterial properties property.

## Conclusion

Hemocompatible PSf membranes with good anticoagulation and antifouling properties were prepared *via* covalently grafting acrylic acid and SHPCS. The –COOH group was successfully grafted onto the surface of the PSf membrane, and SHPCS was also successfully grafted onto the AA-PSf membrane surface in a highly efficient and convenient method, which was confirmed by ATR-FTIR and XPS. The SEM images showed that the membrane after modification exhibited an asymmetric finger-like pore structure, proving that the modification process had no impact on the structure of the pristine membrane. What's more, the hydrophilicity of the membrane improved to a great extent after grafting SHPCS, as confirmed by the water contact angle decreasing from 86° to 22°, and all modified membranes displayed higher blood compatibilities than the pristine PSf membranes, such as lower protein adsorption, suppressed platelet adhesion and deformation, decreased hemolysis ratio as well as prolonged PRT and APTT. In summary, an efficient method was researched to modify the pristine PSf membrane, and the SHPCS-AA-PSf membrane showed great potential to be used as a hemocompatible material, especially in hemodialysis.

## Conflicts of interest

The authors declare no conflicts of interest.

## Supplementary Material
